# Development and double cross-validation of new spot urine sodium equation to predict 24-h urine sodium in the Malaysian population

**DOI:** 10.1186/s41043-021-00232-3

**Published:** 2021-05-31

**Authors:** Fatimah Othman, Rashidah Ambak, Mohd Azahadi Omar, Suzana Shahar, Noor Safiza Mohd Nor, Mohamad Hasnan Ahmad, Muhammad Fadhli Mohd Yusoff, Hasnah Haron, Mohd Fairulnizal Md Noh, Tahir Aris

**Affiliations:** 1 Institute for Public Health, National Institutes of Health, Ministry of Health Malaysia, Shah Alam, Selangor Malaysia; 2 Centre of Healthy Ageing and Wellness, Faculty of Health Sciences, Universiti Kebangsaan Malaysia, Kuala Lumpur, Malaysia; 3 Policy and Strategic Planning Section, Allied Health Science Division, Ministry of Health Malaysia, Putrajaya, Malaysia; 4 Diabetes & Endocrine Unit, Institute for Medical Research, National Institute for Health, Ministry of Health Malaysia, Kuala Lumpur, Malaysia

**Keywords:** Spot urine sodium, Spot urine sodium equation, Sodium monitoring, Malaysia, Double cross-validation, Equation development, 24-h urine sodium

## Abstract

**Background:**

Monitoring sodium intake through 24-h urine collection sample is recommended, but the implementation of this method can be difficult. The objective of this study was to develop and validate an equation using spot urine concentration to predict 24-h sodium excretion in the Malaysian population.

**Methods:**

This was a Malaysian Community Salt Study (MyCoSS) sub-study, which was conducted from October 2017 to March 2018. Out of 798 participants in the MyCoSS study who completed 24-h urine collection, 768 of them have collected one-time spot urine the following morning. They were randomly assigned into two groups to form separate spot urine equations. The final spot urine equation was derived from the entire data set after confirming the stability of the equation by double cross-validation in both study groups. Newly derived spot urine equation was developed using the coefficients from the multiple linear regression test. A Bland-Altman plot was used to measure the mean bias and limits of agreement between estimated and measured 24-h urine sodium. The estimation of sodium intake using the new equation was compared with other established equations, namely Tanaka and INTERSALT.

**Results:**

The new equation showed the least mean bias between measured and predicted sodium, − 0.35 (− 72.26, 71.56) mg/day compared to Tanaka, 629.83 (532.19, 727.47) mg/day and INTERSALT, and 360.82 (284.34, 437.29) mg/day. Predicted sodium measured from the new equation showed greater correlation with measured sodium (*r* = 0.50) compared to Tanaka (*r* =0.24) and INTERSALT (*r* = 0.44), *P* < 0.05.

**Conclusion:**

Our newly developed equation from spot urine can predict least mean bias of sodium intake among the Malaysian population when 24-h urine sodium collection is not feasible.

## Background

Sodium intake is a known risk factor for hypertension, a leading risk factor for cardiovascular disease [1, 2], which bears the highest disease burden in Malaysia. Global projections estimated that 1 in 10 deaths from cardiovascular is attributed to excess sodium intake [3]. Therefore, accurate monitoring is essential to support public health efforts to reduce excess intake and its associated diseases.

Dietary assessments such as 24-h diet recall and food questionnaire have been used to assess dietary sodium intake. However, these methods are known to be inaccurate due to errors in recall and recording even though it has a lower subject burden [4, 5]. The alternative 24-h urine sodium test is considered to be the most valid and reliable method for 24-h sodium estimation [6]. In the presence of complete urine collection and constant sodium intake for several days, 24-h urine reflects about 90% of sodium intake on that day [7].

However, 24-h urine collection is highly burdensome and time- and cost-intensive for a large population study [8]. In efforts to overcome this limitation, several studies have developed an equation based on spot urine and anthropometric measures to estimate 24-h urinary sodium excretion [8–11]. Collecting spot urine specimen is feasible and low cost, but it there is a large diurnal variation in sodium excretion [12–14]. Despite this fact, prediction equations from spot urine have been developed to estimate and monitor sodium intake for population-based studies [8–11, 15].

There are two major approaches for developing the equation derived from spot urine. The first approach is through direct regression of sodium concentration from spot urine and anthropometry measurements on 24-h urine sodium excretion, such as the INTERSALT equation [11, 16]. The second approach, which was developed by Tanaka and Kawasaki, is by multiplying the spot urine sodium to creatinine ratio by the predicted or actual 24-h urine creatinine [8, 10]

At present, the most frequent prediction equations used for monitoring 24-h urine sodium in Asia are Tanaka and Kawasaki’s method. The formation of the equation was based on the assumption that sodium and creatinine ratio from spot urine is proportionate to the one of 24-h urine excretion [8, 10]. However, this equation, which was developed for the Japanese population, has been shown to perform poorly in determining 24-h urine sodium intake among Malaysian adults [17].

To the best of our knowledge, there is no specific equation derived from spot urine to predict 24-h urine sodium for the Asian population by using linear regression statistical test. Furthermore, no specific spot urine sodium equation to estimate 24-h urine sodium has been developed for the Malaysian population. Thus, this study aims to develop and validate a new prediction equation using this method to fill the gap.

## Methods

The spot urine study was a sub-study of the Malaysian Community Salt (MyCoSS) survey, which was a cross-sectional, nationally representative household survey, conducted in all 14 states in Malaysia. Data of 1300 respondents from the MyCoSS were used to estimate the sample size of the present study. Hence, a minimum sample size of 692 from the MyCoSS participants was required to develop and validate the spot urine sodium equation based on the estimated correlation coefficient of 0.15 between spot urine sodium and 24-h urine sodium [17]. Eligible respondents were Malaysian adults aged 18 years and older who were not pregnant, fasting during study, having difficulty to collect 24-h urine, and diagnosed to have kidney disease.

Data was collected between October 2017 and March 2018 and involved 24-h urine and spot urine collections. Urine excretion that was collected in the next 24 hours that ends at the following morning with the first void upon waking was recorded as 24-h urine. Urine excretion, which was collected with the first void on the second morning urine excretion, after completing 24-h urine collection was measured as spot urine. The two urine specimens were collected in separate containers. Out of 1300 - targeted participants, 798 of them completed 24-h urine collection, and 768 of them collected spot urine. A complete 24-h urine collection was defined as (1) total 24-h urinary volume ≥ 500 mL, (2) recorded collection timing of ≥ 20 h, (3) no missing urine, and (4) 24-h urinary creatinine < 6 mmol/day for men and < 4 mmol/day for women [18]. No any criteria were used to evaluate the appropriateness of the spot urine collection.

One-milliliter urine aliquot was taken from the 24-h urine and spot urine for sodium, creatinine, and potassium analysis. In the laboratory, sodium and potassium were determined with an ion-selective electrode method in Architect C, System Analyzer. Creatinine was tested using Kinetic Alkaline Picrate in a similar analyzer.

Body weight and height measurements were measured in duplicate by standardized techniques and calibrated in digital electronic weighing scale (TANITA, HD 319) and SECA Stadiometer 213 (Germany). Other information including age, gender, and socio-demography was obtained through interviews. Permission to conduct this study was obtained from the Medical Research and Ethics Committee (MREC), Ministry of Health Malaysia.

### Development and double cross-validation of spot urine equation to predict 24-h urine sodium excretion

**Fig. 1:**
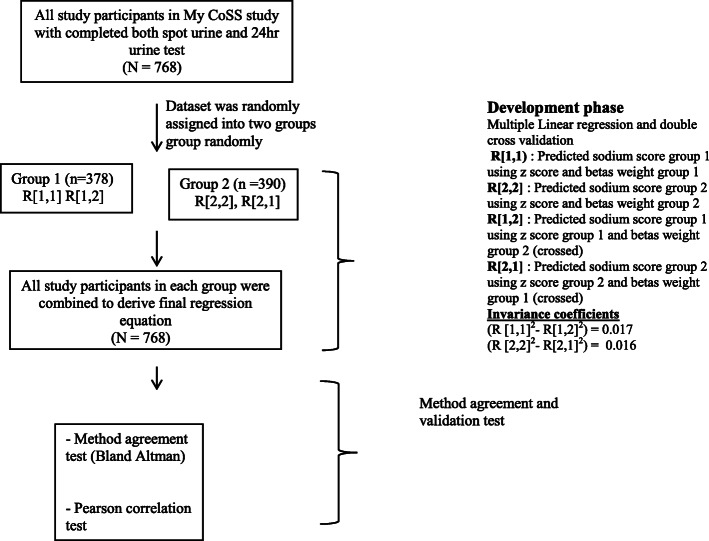
Participant recruitment, development, and validation phase flowchart

In the development phase, the entire data set from the 768 subjects who completed both spot and 24-h urine sodium was randomly split into subgroup 1 and subgroup 2 (Fig. 1). The optimal sample size of each group was confirmed by calculating the sample size for correlation coefficient using MedCalc Statistical Software, version 18.10 [19]. Given type 1 error (alpha) of 0.05, type 11 error (beta) of 0.20, and estimated correlation coefficient of 0.15, the minimum required sample size for each group was 346 respondents.

The equation was developed by using a double cross-validation where an equation was developed for each, with the opposite group being used to cross-validate each equation [20, 21]. In this test, the equation prediction derived from subgroup one was validated against subgroup two, and at the same time, the equation prediction derived in subgroup two was validated against subgroup one. This was accomplished by calculating multiple regression coefficients for both groups: group one R[1,1], R[1,2] and group two R[2,2], R[2,1], where R is multiple regression coefficients computed using each composite z score and beta weight from similar group R[1,1]/ R[2,2] and by crossing beta weight of the other group R[1,2]/ R[2,1] (Fig. 1). Invariance of the results was calculated based on the difference of the squared coefficients between (R[1,1]^2^ − R[1,2]^2^) and (R[2,2]^2^ − R[2,1]^2^). A small difference between squared correlation coefficients (shrinkage) is indicative of stability and replication of the results [20, 21]. Subgroups were then combined and a single equation was developed using the entire data set if the difference of squared correlation coefficients (shrinkage) is small.

### Method agreement and validation of new equation

Bland-Altman plot was calculated to assess the agreement between sodium estimated using predicting equation and those measured by 24-h urine sodium. Other established equations from Tanaka [10] and INTERSALT [11] were assessed using Bland-Altman plot to compare its performance with the new equation. Final correlation coefficients to examine the relation between the predicted equation and measured 24-h urine sodium was determined.

### Statistical analysis

Stepwise multiple linear regression analysis was used to derive a prediction equation for 24-h urine sodium. Measured 24-h urine sodium was the dependent variable and gender, body weight, height, age, spot potassium, spot creatinine, and spot sodium were the predictive variables to form the equation. Variables with 5% level of significance were selected for the final variables to estimate 24-h urine sodium. Regression diagnostic and assumption checking were performed. All statistical analyses were conducted using SPSS version 22 and MedCalc Statistical software version 18.10 [19].

## Results

### Subject characteristics

Recruitment flow of subjects for this study is illustrated in Fig. 1. Socio-demographic characteristic of the 378 subjects in the subgroup 1 and 390 subjects in the subgroup 2 are summarized in Table 1. There was no significant difference of the characteristics between subgroups (*P* > 0.05). Urine test parameters and anthropometry measurements in both subgroups and overall subjects are summarized in Table 2. There was no significant difference in anthropometry components and urine test measurements for sodium, potassium, and creatinine from 24-h urine and spot urine in both subgroups study (*P* > 0.05).

**Table 1:** Socio-demographic characteristic of participants

	Study population (***N*** = 768)	Group 1 (***n*** = 378)	Group 2 (***n*** = 390)	***p*** value
**Sex,** ***n*** **(%)**				
Male Female	340 (42.5) 460 (57.5)	152 (40.2) 226 (59.8)	175 (44.9) 215 (55.1)	0.078
**Strata,** ***n*** **(%)**				
Urban Rural	308 (40.1) 460 (59.9)	158 (41.6) 220 (58.2)	150 (39.5) 240 (61.5)	0.189
**Ethnicity,** ***n*** **(%)**				
Malay Chinese Indian Bumiputra Sabah Bumiputra Sarawak Others	491 (63.9) 81 (10.5) 43 (5.6) 79 (10.3) 62 (8.1) 12 (1.5)	232 (61.4) 42 (11.1) 18 (4.8) 45 (11.9) 34 (9.0) 7 (1.9)	259 (66.4) 39 (10.0) 25 (6.4) 34 (8.7) 28 (7.2) 5 (1.3)	0.410
**Age, mean (sd)**	49.08 (15.1)	48.59 (15.4)	49.55 (14.9)	0.371
**Marital status,** ***n*** **(%)**				
Never married Married Separated Widowed	89 (11.6) 569 (74.1) 23 (3.0) 86 (11.2)	45 (11.9) 276 (73.0) 12 (3.2) 45 (11.9)	44 (11.3) 293 (75.1) 11 (2.8) 42 (10.5)	0.935
**Education attainment,** ***n*** **(%)**				
None Primary education Secondary education Tertiary education	84 (8.3) 160 (20.8) 367 (47.8) 177 (23.0)	33 (8.7) 82 (21.7) 181 (47.9) 82 (21.7)	31 (7.9) 78 (20.0) 166 (47.7) 95 (24.4)	0.788

**Table 2:** Anthropometry and urine parameters of the subjects

	Group 1 (***n*** = 378)	Group 2 (***n*** = 390)	Study population (***N*** = 768)	***P*** value
	Mean (sd)			
Weight (kg)	65.84 (14.01)	67.34 (14.95)	66.60 (14.65)	0.149
Height (m^2^)	1.58 (0.08)	1.58 (0.09)	157.94 (8.74)	
Body mass index (kg/m^2^)	26.48 (5.07)	26.87 (5.51)	26.68 (5.33)	0.303
Total urine volume (mL) (24-h adjusted)	1597.20 (876.38)	1465.85 (804.90)	1563.53 (874.29)	0.030
24-h urine sodium (mg/day)	2721.43 (1212.53)	2610.18 (1128.78)	2694.40 (1269.16)	0.189
24-h urine creatinine (g/day)	1.01 (0.48)	0.99 (0.40)	1.003 (0.439)	0.681
24-h urine potassium (mg/day)	1066.29 (486.38)	1091.11 (560.09)	1078.89 (524.91)	0.513
Spot urine sodium (mg/L)	2013.99 (1292.92)	1986.79 (1263.09)	2002.75 (1279.95)	0.768
Spot urine potassium (mmol/L)	37.60 (29.22)	39.30 (35.12)	37.65 (32.27)	0.464
Spot urine creatinine (mg/dL)	97.55 (68.15)	98.99 (68.99)	96.34 (68.38	0.771

### Development of equation to predict 24-h urine and regression model

The double cross-validation test flow for predicting 24-h urine derived from regression test in both subgroups study is displayed in Fig. 1. Shrinkage power of the equation was small, − 0.016 and 0.017 when each of the derived equations was validated against each other subgroup. This indicates the similarity and stability of the equations as the shrinkage approaches zero. As such, the final equation was derived from the entire data set by combining the subjects both in subgroups 1 and 2. The final predictive equation from regression coefficients is shown in Table 3. The new equation incorporating age, gender, weight, and spot urine tests (potassium, sodium, and creatinine) is as follows:

**Table 3:** Twenty-four hour urine sodium predicting equation

Predicting equation	909.368 + 24.052 (weight in kg) − 0.11 (age^**2**^ in year) + (538.38 if male) + 0.269 (spot sodium in mg/L) − 5.469 (spot creatinine in mg/dL) + 5.541 (spot potassium in mmol/L)		
Parameters	***β***	SE	***P*** value
Weight	0.297	2.674	< 0.001
Age^2^	− 0.138	0.026	< 0.001
Male	0.227	78.053	< 0.001
Spot sodium (mg/L)	0.94	0.032	< 0.001
Spot creatinine (mg/dL)	− 0.32	0.703	< 0.001
Spot potassium (mmol/L)	0.153	1.301	< 0.001

909.368 + 24.052 (weight in kg) − 0.11 (age^2^ in year) + (538.38 if male) + 0.269 (spot sodium in mg/L) − 5.469 (spot creatinine in mg/dL) + 5.541 (spot potassium in mmol/L)

### Method agreement and validation of equation to predict 24-h urine

**Fig. 2:**
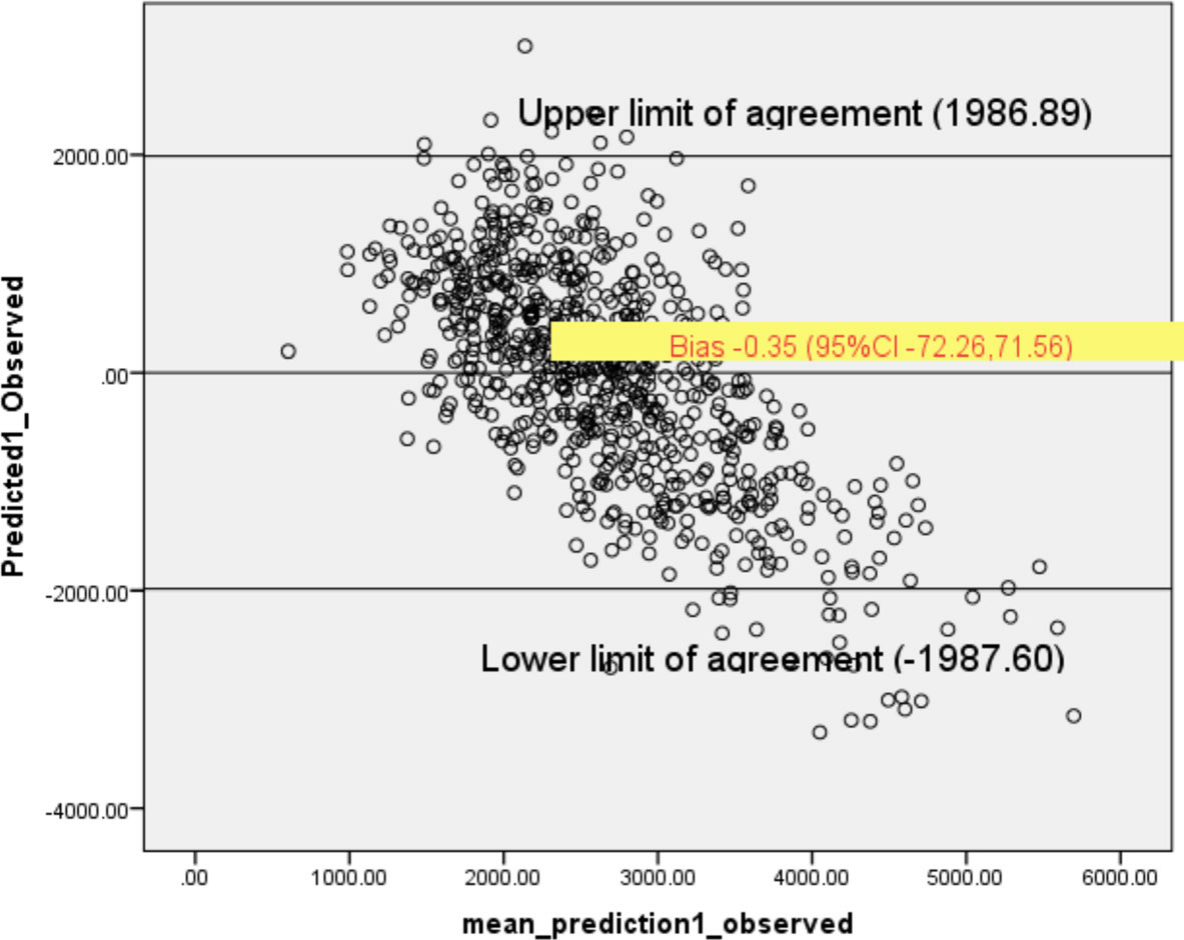
Bland-Altman plot of the mean bias between predicted equation and measured 24-h urine sodium

The initial Pearson correlation for spot urine sodium and 24-h urine sodium was (*r* = 0.219, *P* < 0.01). The correlation strength increased to 0.501 (*P* < 0.01) using the new predicting equation (Table 4). Mean bias (predicted minus measured 24-h urine sodium excretion with the new predicting equation was − 0.35 (− 72.26, 71.56) (*P* > 0.05) and with the INTERSALT was 360.82 (284.34, 437.29) (*P* > 0.05). Using the Tanaka equation, the mean bias was 629.83 (532.19, 727.47) (*P* > 0.05) (Table 5). The upper and lower limit of agreement, mean bias, and the reference line as analyzed in the Bland-Altman plot are displayed in Fig. 2.

**Table 4:** Pearson correlation of measured 24-h urine sodium with spot urine sodium and predicted 24-h sodium from new equation models

	24-h urine sodium (measured)	***P*** value
Spot urine sodium	0.219	< 0.001
New predicted 24-h urine sodium equation	0.501	< 0.001
Tanaka equation	0.232	< 0.001
INTERSALT equation	0.400	< 0.001

**Table 5:** Mean bias limit of agreement between 24-h urinary sodium and new predicting equation, TANAKA and INTERSALT equations

Equation	Mean bias (95% CI)	Upper limit	Lower limit
Predicting equation	− 0.35 (− 72.26, 71.56)	1986.89	− 1987.60
Tanaka^1^	629.83 (532.19, 727.47)	3327.99	− 2068.33
INTERSALT^2^	360.82 (284.34, 437.29)	2473.99	− 1752.35

^1^Tanaka, equation (2.54 ÷ 1000 × 23 × [spotNa (mmol/L)/(spotCr (mg/dL) × 10] × [− 2.04 × age (years) + 14.89 × weight (kg) + 16.14 × height (cm) − 224.45]}^0.392^

^2^INTERSALT, equation (male: 2.54 ÷ 1000 × 23 {25.46 + [0.46 × spotNa (mmol/L)] − [2.75 × spotCr (mmol/L)] − [0.13 × spot K (mmol/L)] + [4.10 × BMI ] + [0.26 × age]. Female: 2.54 ÷ 1000 × 23 {5.07 + [0.34 × spotNa (mmol/L)] − [2.16 × spotCr( mmol/L)] − [0.09 × spot K (mmol/L)] + [2.39 × BMI ] + [2.35 × age − [0.03 × age^2^ (years)]

## Discussion

A simple equation to estimate 24-h urine sodium in the Malaysian adult population was developed in this study. The prediction equation moderately correlated (*r* = 0.5, *P* < 0.01) with measured 24-h urine sodium. When used with spot urine specimen and other parameters including weight, age, and gender, this prediction equation may provide the biased level of population mean sodium compared with other established equations, namely Tanaka’s [10] and INTERSALT [11]. The performance of the Tanaka equation in this study showed the greatest mean bias compared to the current predicting equation and the INTERSALT. A validation study using Tanaka’s equation to estimate 24-h sodium intake among health staff in Malaysia has been observed to overestimate the actual sodium intake in that study population [22]. This is possibly due to its development equation, which was based on the multiplication of estimated urine creatinine and the ratio of spot urine sodium and creatinine. Based on Tanaka’s, the excretion of 24-h creatinine was equivalent to the estimated creatinine excretion, as it is the end product of creatinine metabolism in the muscle, which is excreted constantly through the kidney. However, data observed in this study showed only a weak correlation between spot urine creatinine and 24-h urinary creatinine (*r* = 0.28), suggesting a variable urine creatinine excretion in relation to age, muscle mass, and dietary factors such as meat consumption [23, 24].

The INTERSALT equation when compared to Tanaka’s has produced a lesser mean bias and a comparable performance in Pearson correlation with the new prediction equation. The INTERSALT development equation was derived using regression analysis, which include the significant independent parameters in spot urine and subjects characteristics that predict 24-h urinary sodium [11]. This approach was found to give a better estimation and stronger correlation with measured 24-h urine sodium as observed in this study.

Despite the least mean bias of predicted 24-h urinary sodium derived from the new equation, the predicted sodium was not consistent across low to high level sodium and tended to underestimate high sodium excretion. Compared to sodium excretion, this new equation could underestimate individual sodium intake as much as 3000 mg/day. Underestimation was also reported in other studies by as much as 7000 mg/day using the spot urine equations [25, 26].

The Bland-Altman plot in Fig. 2 showed the predictive equation tended to underestimate sodium excretion at 3000 mg or above. Although there was no significant systematic difference as the line of equality was within the limit of agreement, caution is needed when evaluating sodium excretion across high sodium intake among the individual intake with more than 3000 mg/day). A similar pattern was also observed using another spot urine equation to estimate 24-h urine sodium [11, 15].

The possible reason on why the spot urine equation underestimated high sodium concentration might be due to the diurnal variability in sodium excretion per day [14, 27]. Sodium excretion can be altered by position, exercise, diet, and hemodynamic factors [7, 12, 28]. In this study, spot urine, which was part of the total urine excretion, was taken only once in the early morning. In addition, sodium concentration has been observed to be lowest in the early morning and highest at mid-day [29]. This observation might support the lower sodium level predicted by our equation compared to measured 24-h urine when as the single spot urine was collected in the morning. When using spot urine collected in the morning, afternoon, or evening, the INTERSALT equation in another study appeared to estimate group mean sodium level [30].

There may be some limitations in this study because of the single collection and timing of spot urine. In addition, this equation also has not been validated to the external population. However, the strengths of this study are its a nationwide sampling that represents the populations with various lifestyles and environmental and dietary intake characteristics. We are also able to limit the external factor leading to the inadequacy of 24-h urine collection and exclude potential participants that might affect 24-h urine sodium excretion.

## Conclusions

In conclusion, the spot urine equation developed in this study was able to estimate sodium intake for the Malaysian population. Although the use of 24-h urine sodium excretion is the recommended test to measure general mean population sodium intake, the findings from the predictive equation tailored to the Malaysian population will facilitate the national surveillance efforts to chart mean trends in sodium intake.

## Data Availability

The data sets used and/or analyzed during the current study are available from the corresponding author on reasonable requests.

## Abbreviations

MyCoSS: Malaysian Community Salt Study
24-h: 24-hour
BMI: Body mass index
WHO: World Health Organization
